# Biochemical and transcriptomic evaluation of a 3D lung organoid platform for pre-clinical testing of active substances targeting senescence

**DOI:** 10.1186/s12931-023-02636-7

**Published:** 2024-01-03

**Authors:** Michelle Brand, Felix Ritzmann, Kathrin Kattler, Deivydas Milasius, Yiwen Yao, Christian Herr, Susanne H. Kirsch, Rolf Müller, Daniela Yildiz, Robert Bals, Christoph Beisswenger

**Affiliations:** 1 Department of Internal Medicine V – Pulmonology, Allergology and Critical Care Medicine, Saarland University, 66421 Homburg, Germany; 2 Department of Drug Delivery (DDEL), Helmholtz-Institute for Pharmaceutical Research Saarland (HIPS), Helmholtz Centre for Infection Research (HZI), Saarland University Campus, 66123 Saarbrücken, Germany; 3 Department of Genetics/Epigenetics, Saarland University, 66123 Saarbrücken, Germany; 4 Department of Microbial Natural Products (MINS), Helmholtz Institute for Pharmaceutical Research Saarland (HIPS), Helmholtz Centre for Infection Research (HZI), Saarland University Campus, 66123 Saarbrücken, Germany; 5 Department of Pharmacy, Saarland University, 66123 Saarbrücken, Germany; 6 Experimental and Clinical Pharmacology and Toxicology, PZMS, and Center for Human and Molecular Biology (ZHMB), Saarland University, 66421 Homburg, Germany

**Keywords:** Lung diseases, Organoid, Senescence, Toxicity, Inflammation, Preclinical studies, Single cell sequencing

## Abstract

Chronic lung diseases such as chronic obstructive pulmonary disease and cystic fibrosis are incurable. Epithelial senescence, a state of dysfunctional cell cycle arrest, contributes to the progression of such diseases. Therefore, lung epithelial cells are a valuable target for therapeutic intervention. Here, we present a 3D airway lung organoid platform for the preclinical testing of active substances with regard to senescence, toxicity, and inflammation under standardized conditions in a 96 well format. Senescence was induced with doxorubicin and measured by activity of senescence associated galactosidase. Pharmaceutical compounds such as quercetin antagonized doxorubicin-induced senescence without compromising organoid integrity. Using single cell sequencing, we identified a subset of cells expressing senescence markers which was decreased by quercetin. Doxorubicin induced the expression of detoxification factors specifically in goblet cells independent of quercetin. In conclusion, our platform enables for the analysis of senescence-related processes and will allow the pre-selection of a wide range of compounds (e.g. natural products) in preclinical studies, thus reducing the need for animal testing.

**Supplementary Information:**

The online version contains supplementary material available at 10.1186/s12931-023-02636-7.

## Background

Organoids are self-assembling structures that differentiate from progenitor cells and mimic various aspects of organs such as architecture, cellular composition and function. Human organoid models have enormous potential in basic research, pre-clinical studies, therapy development and personalized medicine [1, 2].

The airway epithelium extends from the upper airways to the respiratory bronchioles. It is composed primarily of basal, secretory, club, goblet and ciliated cells as well as rare cells such as tuft cells and ionocytes [3]. As progenitor cells, basal cells can be cultivated from human tissue sampling and differentiate within 30 days into airway (bronchial) organoids, in which different cell types such as basal, secretory, goblet and ciliated cells can be distinguished [4–6]. In these organoids, it is possible to depict individual processes of disease development. For instance, stimulation with IL-13 or bacterial-derived flagellin during the differentiation phase initiates goblet cell metaplasia which is characteristic for obstructive pulmonary diseases [4, 5].

Chronic lung diseases such as chronic obstructive pulmonary disease and cystic fibrosis are not curable or easily treatable. Therapeutic interventions are mostly symptomatic (e.g. eradication therapy for secondary infections, bronchodilators), but in the rarest of cases (e.g. gene therapy) they are causal [7]. Chronic lung diseases are associated with persistent inflammation that leads to damage to the epithelium and loss of lung structure and function. Cellular senescence, a state of cell cycle arrest associated with epithelial dysfunction, likely contributes to the progression of chronic lung diseases. Studies showed that senescent cells are more common in the lungs of patients with chronic lung disease [7–9]. Senescence markers are upregulated in pneumocytes from IPF and COPD patients and pre-clinical studies suggest that senescent pneumocytes may have a function in chronic lung diseases [10–12]. Preclinical studies in mice suggest that senescent airway epithelial cells promote chronic lung inflammation and that pharmacological reduction in the number of senescent cells attenuates pulmonary inflammation [10, 13]. Lung epithelial cells are therefore an interesting target for therapeutic interventions aimed at silencing or eliminating senescent cells. Natural products derived from plants, fungi or bacteria including already market approved extracts that are well tolerated are here of particular interest [14, 15].

Numerous animal experiments are carried out in aging and senescence research [16]. In the development and safety assessment of new drugs, extensive in vivo studies are often necessary and mandatory according to the applicable guidelines before approval. However, animal experiments are associated with considerable animal consumption and suffering. In addition, the results obtained are often not extrapolatable to humans due to species-specific divergences in the physiology of humans and animals, resulting in a low success rate in drug development [17, 18]. It is therefore imperative to develop adequate models based on human cells that reduce the need for animal experiments and, in the best case, replace them entirely. Here we present an airway organoid-based assay that allows the analysis of therapeutics for epithelial senescence as well as associated toxicity and inflammation. Our motivation behind the development of the organoid assay is the future use of this novel analytical platform to test potential therapeutic agents such as natural products, novel natural products of microbial origin, anti-inflammatory drugs or already approved phytoextracts with respect to their influence on cell senescence, toxicity and inflammatory processes of the lung epithelium via standardized screening methods. In the future, this could help find treatment options for chronic lung diseases without having to carry out animal experiments.

## Materials and methods

### Organoid culture

Primary human epithelial cells (HBECs) isolated from bronchial brushings during routine examinations were cultured in the presence of 3T3 feeder cells as described before [19]. Passage 1 cells were differentiated to bronchospheres as described before [4, 5]. Cells from a total of 14 different donors were used for the study. The protocol was approved by the Institutional Review Board (ethics committee) of the Saarland State Medical Association, and informed consent was obtained from the patients. Briefly, 40 µL of a 25% Matrigel solution (Corning, USA) in differentiation media (DM, Lonza, Switzerland), mixed in equal volumes with DMEM-F12, Gibco, USA) with 50 nM retinoic acid were added to each well of a 96-well plate. A solution of 80 µL HBECs (3 × 10^4^ cells per mL, passage 1 to 2) in DM (5% Matrigel, 50 nM retinoic acid) were added to each well. Two days after seeding the HBECs, media was replaced every 4 days, Phase contrast images of each well were taken (Axiovert 25, Zeiss, Germany) and the presence of lumina was documented.

### Senescence assay, MTT, LDH, and measurement of cytokines

Organoids were used for experiments 21 to 30 days after seeding. Nine wells per group were stimulated with doxorubicin (100 nM), quercetin (50 µM), dasatinib (200 nM), the combination of doxorubicin with quercetin or dasatinib, or control medium (DM) for 48 h. To test whether quercetin treatment does revert doxorubicin-induced senescence, organoids were incubated with doxorubicin or control medium for 48 h. The organoids were then incubated for a further 48 h with doxorubicin, quercetin, the combination of doxorubicin and quercetin, or with control medium. The supernatant of each well was collected and kept at -80 °C for further analysis. Organoids were removed from the Matrigel prior to senescence and MTT assays. 200 µL of cold Cell Recovery Solution (Biotium, USA) were added to each well. After 10 min incubation at 4 °C, the organoids were collected (3 wells of each condition were pooled). The organoids were resuspended in 100 µl DM and transferred into a black 96-well plate with clear bottom. Activity of ß-galactosidase was measured using the Senescence Assay Kit (Abcam, UK) adapted for detection in plate reader. For this purpose, 100 µl of DM with senescence dye (6 µl per 1000 µl) were added to each well. The organoids were incubated at 37 °C for 2 h. The organoids were then washed twice with washing buffer and taken up in 200 µl washing buffer. Fluorescence was measured at 485 nm excitation and 520 nm emission using a microplate reader (FLUOstar Omega, BMG Labtech, Germany). The metabolic activity of the organoids was measured with the MTT cell viability assay kit (Biotium, USA) according to the manufacturer’s protocol after removing Matrigel. Lactate dehydrogenase (LDH) was measured in supernatants using an LDH assay kit (Abcam, #65,393, UK) according to the manufacturer’s protocol.

Concentrations of inflammatory markers were measured in supernatants by enzyme-linked immunosorbent assay (ELISA, R&D Systems, Germany) or magnetic luminex assay (R&D Systems, Minneapolis, MN, USA) using MAGPIX (Luminex corporation, Austin, USA) according to the manufacturer’s protocol.

Basal cells cultured in black 96-well plates were stimulated with doxorubicin (100 nM), quercetin (50 µM), dasatinib (200 nM), the combination of doxorubicin with quercetin or dasatinib, or control medium for 48 h (three wells per group). Activity of ß-galactosidase was measured directly as described above.

### Single-cell RNA sequencing (scRNA-seq)

Cell and library perparation. For scRNA-Seq, organoids out of 10 wells of each group were collected by removing Matrigel with cold Cell Recovery Solution (Corning, #354,253). The organoids were washed in cold PBS and dissociated with TrypLE Express (Gibco) under gentle resuspension for 15 min to generate a single cell solution. After washing the cells in PBS to remove TrypLE Express, the cell solutions per condition were pooled with the BD™ human Single-Cell Sample Multiplexing Kit (#633,781) and processed as indicated in the manufacturer’s protocol. The cell number as well as the viability was determined using DRAQ7 (ThermoFisher, #15,106) and calcein (BD Biosciences, #564,061). Cell were captured on the BD Rhapsody System and sequencing libraries were generated using the BD cDNA (#633,773) and WTA Amplification Kit (#633,801) according to the manufacturer’s protocol. Concentration and quality of generated libraries were determined by Qubit Fluoromenter (Qubit dsDNA HS Kit, #Q32851) and Agilent 2100 Bioanalyzer (High sensitivity DNA Kit, #5067 − 4626). The libraries were sequenced on the Novaseq 6000 platform with 50,000 reads per cell.

Data processing and analysis. Raw fastq reads were processed with the BD Rhapsody™ WTA Analysis Pipeline on the Sevenbridges platform, aligning the reads to the human GRCh38 reference genome. Data was analyzed using the Seurat 4.0 R package [20]. Multiplets and undetermined counts were excluded as well as cells with < 1000 detected genes and > 50% mitochondrial genes. The filtered dataset was normalized and scaled by using Seurat NormalizeData (scale factor 10,000) and ScaleData function. Organoids from two different cohorts were combined using the seurat integration method. Cell clusters were identified using a shared nearest neighbors (SNN)-based algorithm (resolution was set to 0.3). Nonlinear dimensional reduction was performend to generate Uniform Manifold Approximation and Projection (UMAP) plots as illustrated. Cluster specific gene markers were identified by Wilcoxon rank sum testing, used by Seurats FindMarker and FindAllMarkers function. Clusters were annotated using known marker genes for the specific cell types [21]. Transcription factor regulon analysis was achieved with the python implementation of SCENIC pySCENIC (version 0.12.0, https://github.com/aertslab/pySCENIC) based on CPM normalized counts of quality filtered single cells. Adjacencies were calculated using pyscenic grn and motifs were pruned with pyscenic ctx against hg38_refseq-r80_10kb_up_and_down_tss.mc9nr.genes_vs_motifs.rankings.feather and hg38_refseq-r80_500bp_up_and_100bp_down_tss.mc9nr.genes_vs_motifs.rankings.feather databases. Then pyscenic AUCell was used to obtain regulon activity scores which were visualized using scanpy (version 1.9.1) [22]. For pathway enrichment analysis, Seurat’s DEenrichRplot command was performed. The enrich.database was set to “KEGG_2019_Human” (max. genes 2000). Splicing kinetics were calculated using velocyto (version 0.17.17) and RNA velocity was modelled with scvelo (version 0.2.4) applying a stochastic model using a distance based KNN graph with default parameters [23, 24].

### Statistical analysis

Comparisons were tested by parametric or nonparametric tests (Student t-test, one-way ANOVA with Tukey’s post hoc test, Mann-Whitney) using the software Prism (GraphPad Software, San Diego, CA). The results were considered statistically significant for p < 0.05. For details, see figure legends. Compositional changes in single cell RNA-seq cluster proportions were calculated using a Bayesian model implemented in scCODA [25] which can handle the low number of replicates (n = 2). The analysis was performed as suggested in the scCODA guidelines. The false discovery rate cut-off (FDR) for selection of credible effects based on their inclusion probabilities was set to 0.4. To ensure robustness of this approach and to exlucde false positive effects, the analysis was independently repeated 10 times. Only those compositional changes that were detected as credible effect in the Bayesian framework in 10 out of 10 runs were reported as significant.

## Results

### Bronchosphere senescence assay

**Fig. 1:**
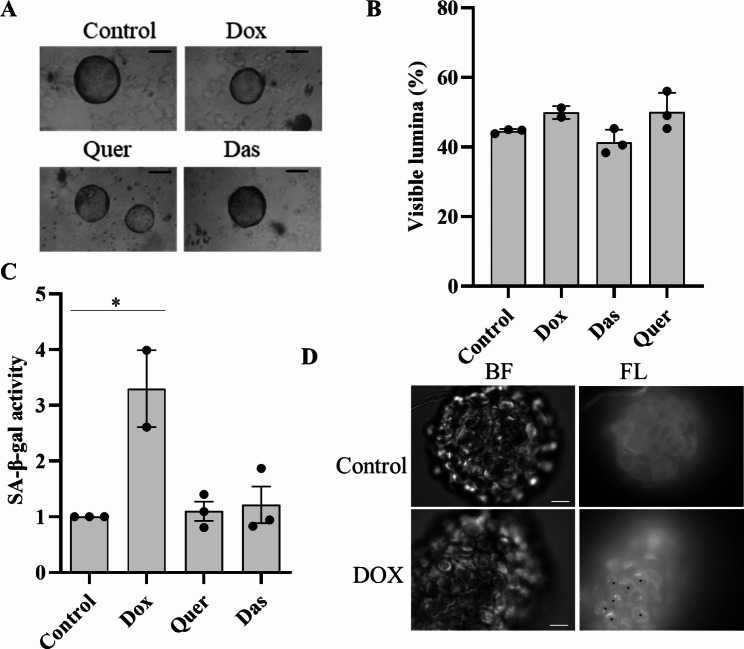
Doxorubicin induces a senescence-like phenotype in 3D bronchial organoids. (**A**) Representative phase contrast images of organoids (scale bar = 100 μm), (**B**) percentage of organoids with visible lumen, and (**C**) senescence associated β-galactosidase (SA-β-gal) activity 48 h after treatment with doxorubicin (Dox), quercetin (Quer), and dasatinib (Das). Each data point represents an independent experiment using cells from a separate donor. Data were compared by one-way ANOVA with Tukey’s multiple comparisons post-test and are shown as the mean ± SEM. *p < 0.05 (**D**) Fluorescence microscopy of organoids 96 h after treatment with Dox (BF = brightfield, scale bar = 50 μm, * indicate SA-β-gal activity)

The topoisomerase II inhibitor doxorubicin (Dox) is widely used to induce cellular senescence [26] whereas the pharmaceutical compounds quercetin (Quer, a plant derived flavonoid natural product) and dasatinib (Das, a synthetic protein kinase inhibitor) have been shown to reduce senescence in various pre-clinical models [27, 28]. First, we tested whether Dox, Quer, and Das change the senescence stage in our lung organoid model. Primary basal cells were differentiated in Matrigel for 21 days. As quality control, formation of lumina was monitored under the phase contrast microscope. Only cultures in which at least 35% of the organoids had a visible lumen, corresponding to greater than 90% lumen formation as detected by H&E staining, were included in the experiments (S1) [5]. The incubation of the organoids with Dox, Quer and Das for 48 h did not result in any visible impairment of the integrity of the organoids (Fig. 1A and B). The senescence associated β-galactosidase (SA-β-gal) is a marker of cellular senescence [27]. Treatment with Dox resulted in a significantly increased activity of SA-β-gal (Fig. 1C). In contrast, treatment with Quer and Das had no significant effect on SA-β-gal activity compared to control. Fluorescence microscopy of organoids loaded with senescence dye also revealed increased SA-β-gal activity in Dox-treated organoids (Fig. 1D). Flow cytometry confirmed the Dox-induced enhanced SA-β-gal activity (S2).

### Exemplary testing of active ingredients in the doxorubicin-induced senescence assay

**Fig. 2:**
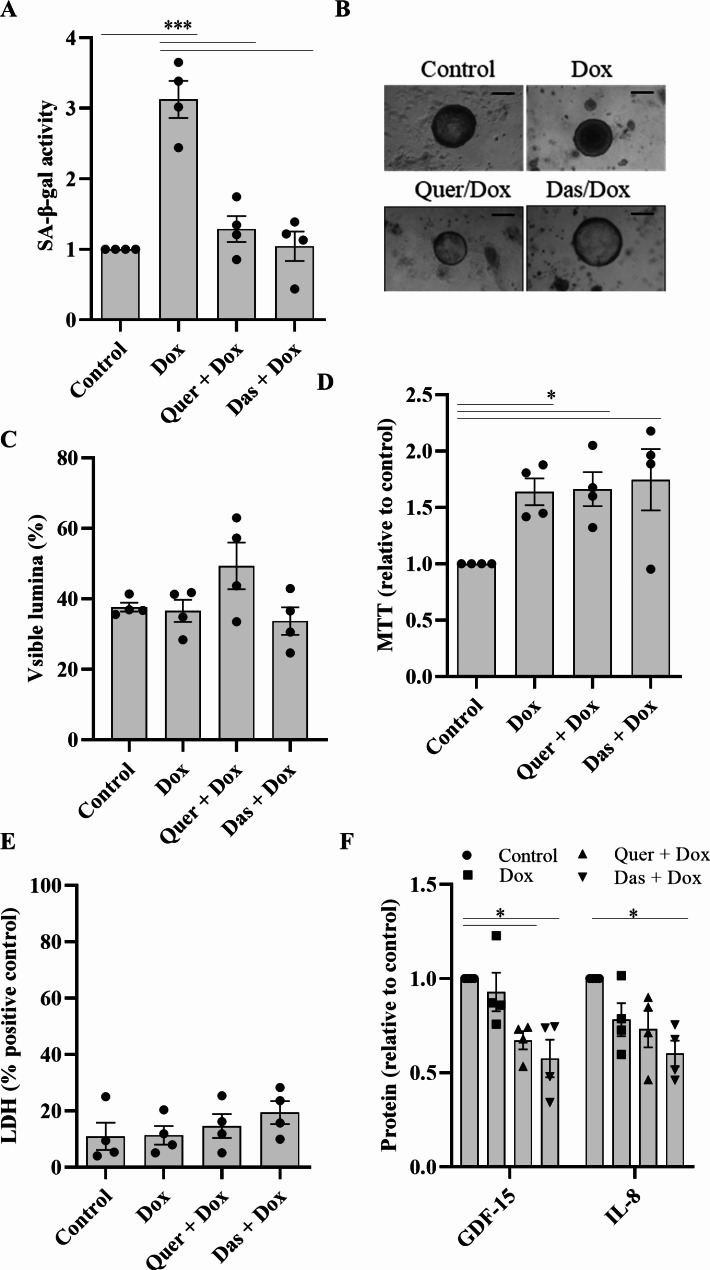
Quercetin and dasatinib counteract doxorubicin-induced senescence. (**A**) SA-β-gal activity, (**B**) representative phase contrast images of organoids (scale bar = 100 μm), (**C**) percentage of organoids with visible lumen, (**D**) metabolic activity, (**E**) release of LDH, and (**F**) release of GDF-15 and IL-8 after 48 h treatment with Dox and the combination of Dox with Quer or Das (relative to control). Each data point represents an independent experiment using cells from a separate donor. Data were compared by one-way ANOVA with Tukey’s multiple comparisons post-test and are shown as the mean ± SEM. *p < 0.05, **p < 0.01 and ***p < 0.001

Next, we tested whether the organoid-based senescence assay can be used for testing of pharmaceutical active ingredients. The organoids were treated with Dox alone or in combination with Quer or Das for 48 h. Quer or Das treatment significantly decreased Dox-induced SA-β-gal activity (Fig. 2A), accompanied by reduced Dox-induced p21 protein expression (S2 B/C). The cyclin-dependent kinase inhibitor p21 promotes arrest of the cell cycle and is a central mediator of cellular senescence [26, 29]. Observation under phase contrast showed that neither Dox alone nor the combination of Dox with Quer or Das resulted in loss of organoid integrity (Fig. 2B and C). Incubation with Dox resulted in an approximately 1.5-fold increased metabolic activity as indicated by MTT test that was not significantly affected by Quer or Das (Fig. 2D). There were no significant differences in the release of LDH in the supernatant between the different treatments (Fig. 2E). As Metformin (Met) has been described to modulate senescence [30, 31] we also tested the effect of Met on Dox-induced senescence. There was no significant effect of Met on Dox-induced SA-β-gal activity (S3A). Met also did not significantly affect the number of organoids with lumina, metabolic activity and release of LDH (S3B to D).

Inflammation is an undesirable side effect of drugs and often associated with senescence. Therefore, we further analyzed the release of inflammatory mediators into supernatants [29]. Treatment of organoids with Dox for 48 h had no significant impact on the background release of the inflammatory cytokine IL-8 and growth/differentiation factor 15 (GDF-15), a factor associated with senescence [29]. However, release of GDF-15 was reduced by additional treatment with Quer or Das (Fig. 2F).

### Quercetin decreases SA-β-gal activity after senescence is induced

**Fig. 3:**
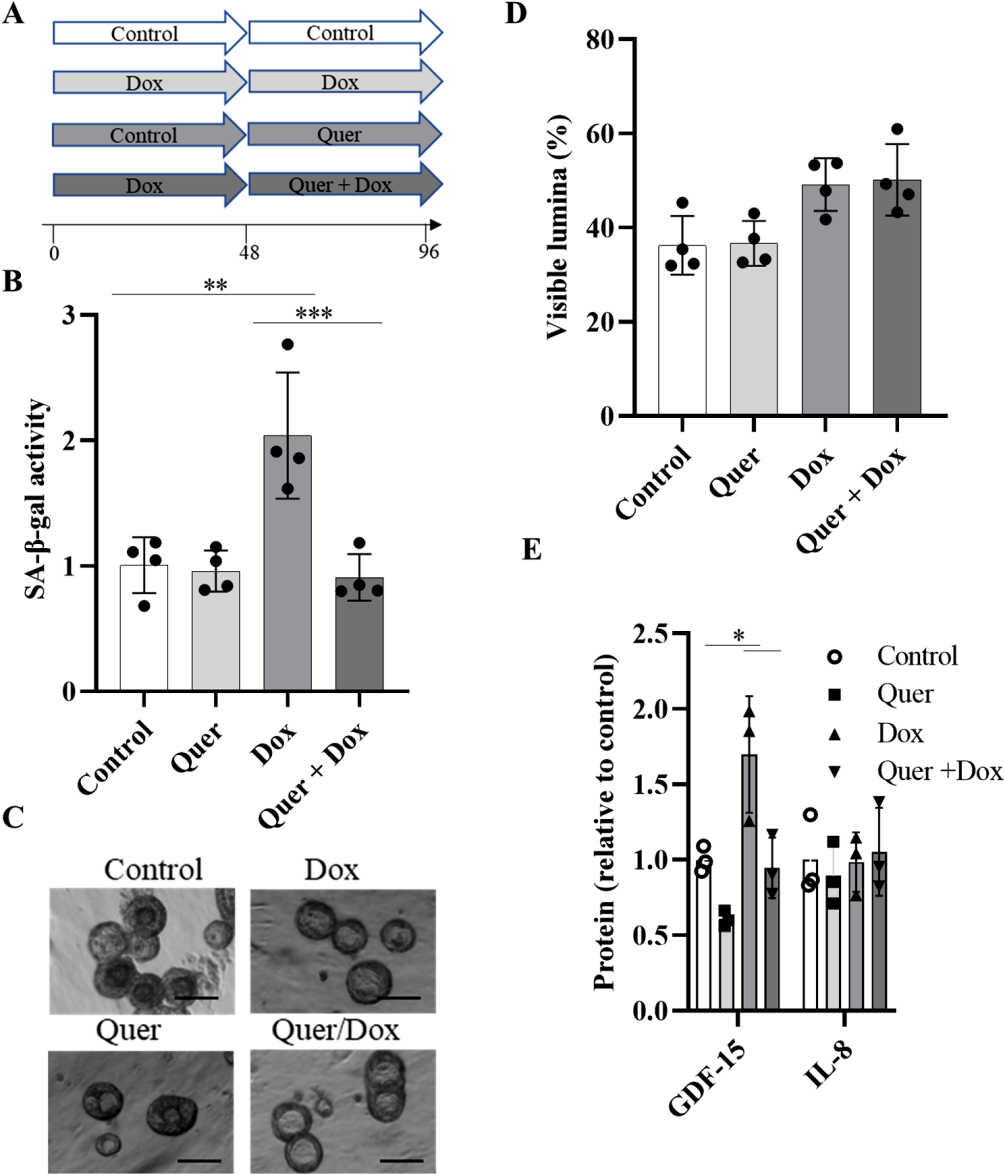
Quercetin decreases SA-β-gal activity after senescence has been induced. (**A**) Schema of the experimental protocol. Organoids were pre-treated with Dox or control medium for 48 h. The organoids pre-incubated with control medium were treated with control medium or Quer, the organoids pre-incubated with Dox were treated with Dox or the combination of Quer and Dox for additional 48 h. (**B**) SA-β-gal activity, (**C**) representative phase contrast images of organoids (scale bar = 50 μm), and (**D**) percentage of organoids with visible lumen. (**E**) Concentrations of GDF-15 and IL-8 in supernatants. Presented data are representative for two independent experiments. Data were compared by one-way ANOVA with Tukey’s multiple comparisons post-test and are shown as the mean ± SD. **p < 0.01 and ***p < 0.001

We tested whether Quer treatment does not only inhibit development of senescence but could also revert these processes. For this, we pre-treated organoids with Dox or control medium for 48 h. The organoids pre-incubated with control medium were subsequently treated with control medium or Quer, whereas the organoids pre-incubated with Dox were treated with Dox or the combination of Quer and Dox for additional 48 h (Fig. 3A). Treatment with Quer significantly reduced Dox-induced SA-β-gal activity (Fig. 3B). The different treatments did not significantly affect the integrity of the organoids (Fig. 3C and D). Release of GDF-15 was significantly reduced by Quer (Fig. 3E).

### Single cell sequencing reveals cells expressing senescence markers

**Fig. 4:**
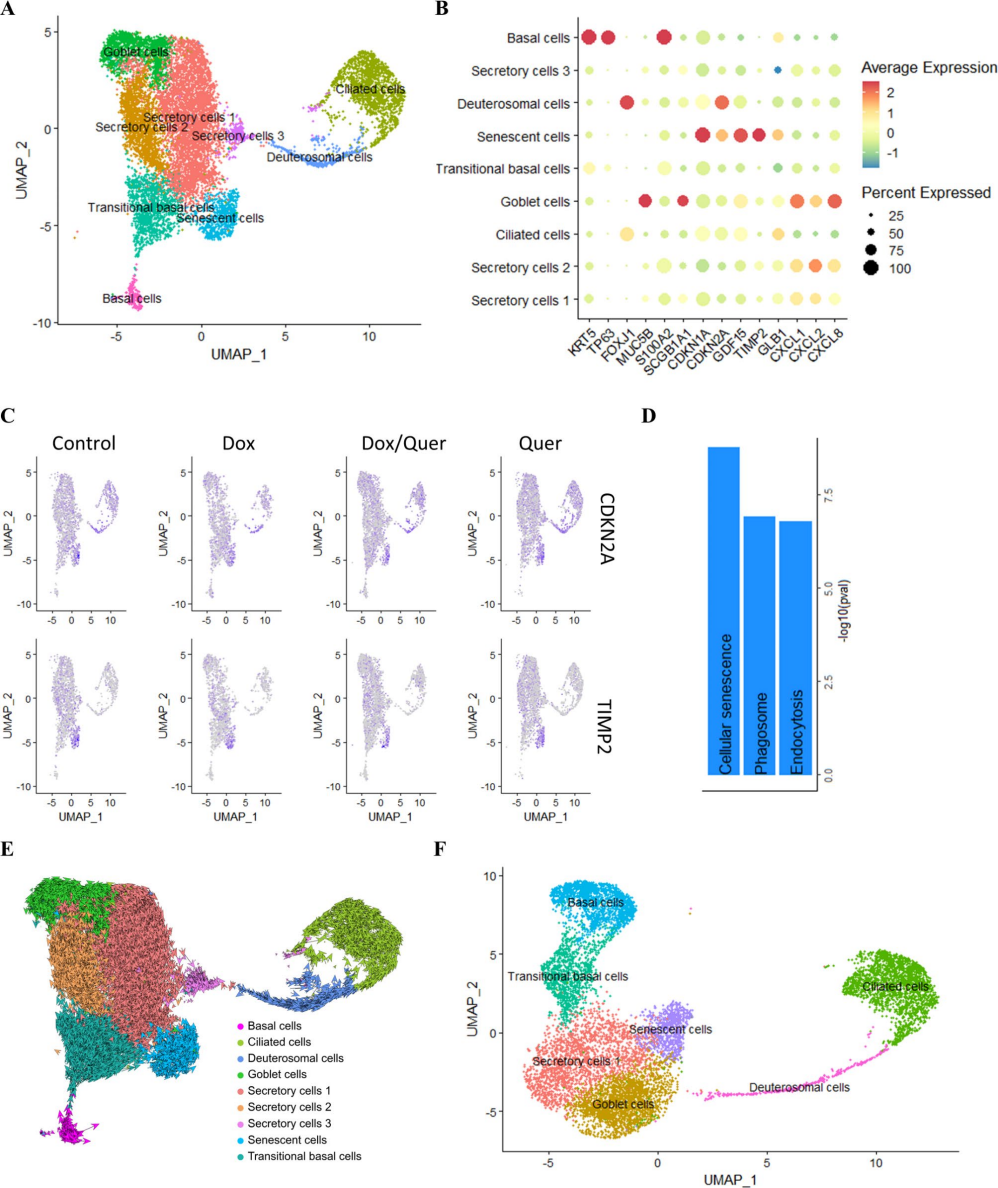
scRNA-seq analysis reveals distinct epithelial cell populations. (**A**–**D**) Organoids were pre-treated with Dox or control medium for 48 h. The organoids pre-incubated with control medium were treated with control medium or Quer, the organoids pre-incubated with Dox were treated with Dox or the combination of Quer and Dox for additional 48 h. (**A**) UMAP visualization of major epithelial cell types. (**B**) Dot plot showing expression of epithelial cell type markers. (**C**) UMAP visualization of cell markers for senescence. (**D**) Bar plot of Hallmark gene set enrichment analysis (KEGG_2019_Human, two-sided Wilcoxon Rank Sum) for pathways enriched in senescent cells. (**E**) RNA velocity analysis of scRNA-seq data. (**F**) UMAP visualization of major epithelial cell types of combined data sets

Next, we analyzed how Dox and Quer affect organoids using scRNA-seq. For this we treated organoids derived from two different donors as shown in Fig. 3A. Representative markers were used to annotate clusters [3, 32]: Basal cells, secretory cells, goblet cells, secretory, deuterosomal and ciliated cells (Fig. 4A and B, S4). Remarkably, we identified a cluster, here defined as senescent cells, with high expression for the common senescence markers *CDKN1A* (p21), *CDKN2A* (p16), *TIMP2*, and *GDF15* [29, 33] (Fig. 4B to C, S4). Pathway enrichment analysis also showed enrichment of the cellular senescence pathway in the senescent cell cluster (Fig. 4D). RNA velocity analysis showed that basal cells differentiate via transitional basal cells to secretory cells as well as via deuterosomal cells to ciliated cells, which correctly reflects the normal differentiation of the airway epithelium (Fig. 4E) [3]. Moreover, transitional basal cells and secretory cells 2 also showed velocity towards senescent cells, which indicates that senescent cells are derived from cells in transition. We also combined these single cell data with another own independent data set containing untreated organoids from 5 donors. The cluster with senescence-like cells was also present in these organoids (Fig. 4F, S5, S6).

Because TIMP2 was almost exclusively expressed in the senescence cluster, we stained organoids for p21 and TIMP2 (S7A). Dox treatment resulted in increased number of p21-positive nuclei and TIMP2 expression (S7 B/C). Increased staining for TIMP2 associated with p21 positive nuclei.

We also performed a regulon analysis using SCENIC to gain insight into transcriptional regulatory networks (S5) [34]. The activity of the SPDEF regulon was increased in secretory cells, SPDEF has been shown to regulate *MUC5B* in airway epithelial cells [35]. The acitivity of the FOXJ1 regulon was increased in ciliated cells, the SNAI2 regulon in basal cells (S8). Senescent cells were marked by an increased activity of the BATF and PRDM1 regulons. BATF and PRDM1 have been shown to limit cell self-renewal and have a function in maintaining developmental stages [36–38].

### Quercetin decreases cells expressing senescence markers

**Fig. 5:**
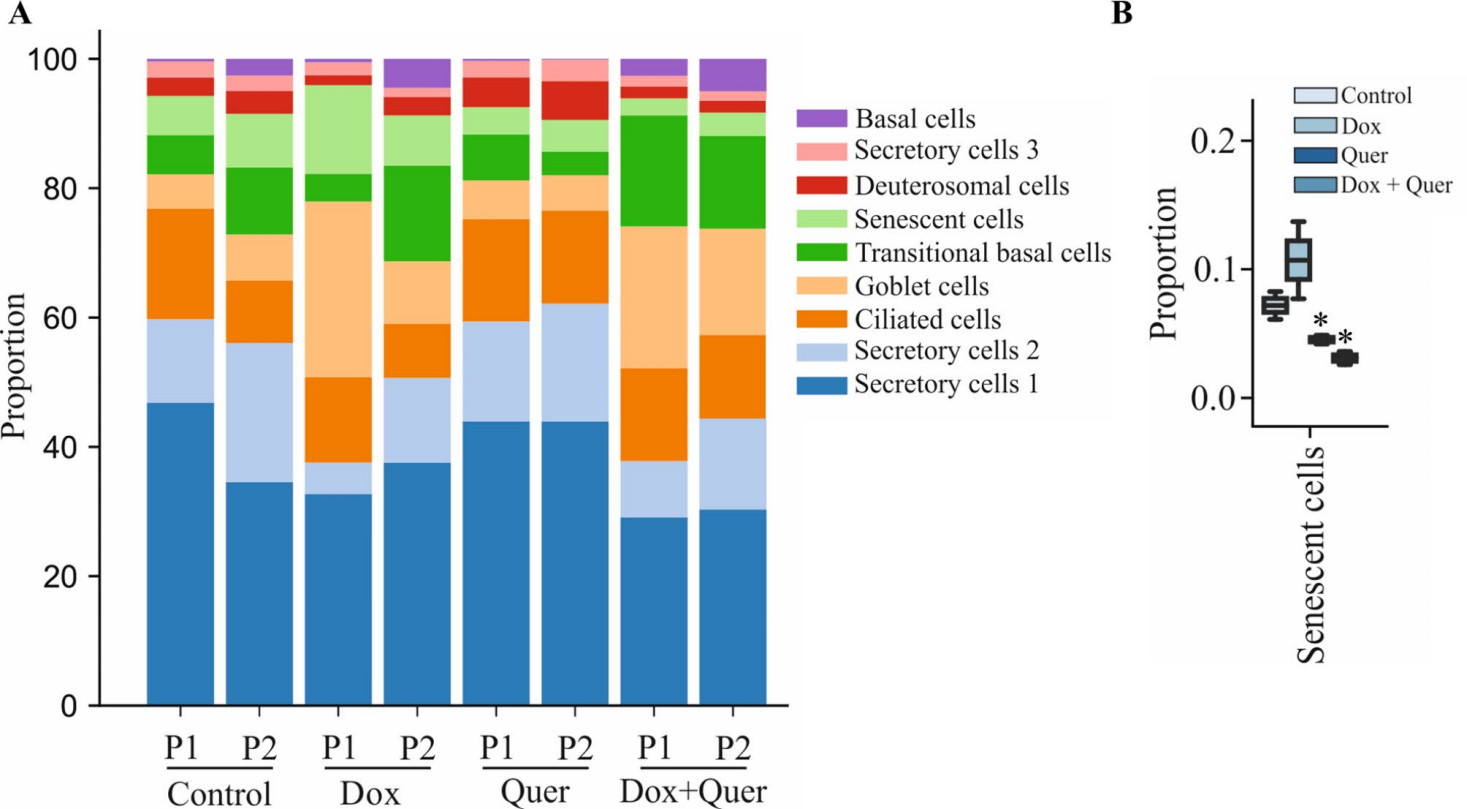
Quercetin decreases the number of cells expressing senescence markers. Organoids were pre-treated with Dox or control medium for 48 h. The organoids pre-incubated with control medium were treated with control medium or Quer, the organoids pre-incubated with Dox were treated with Dox or the combination of Quer and Dox for additional 48 h. (**A**) Proportion of the different cell clusters. (**B**) Proportion of the senescent cell cluster among all clusters. Data were compared by a Bayesian model

**Fig. 6:**
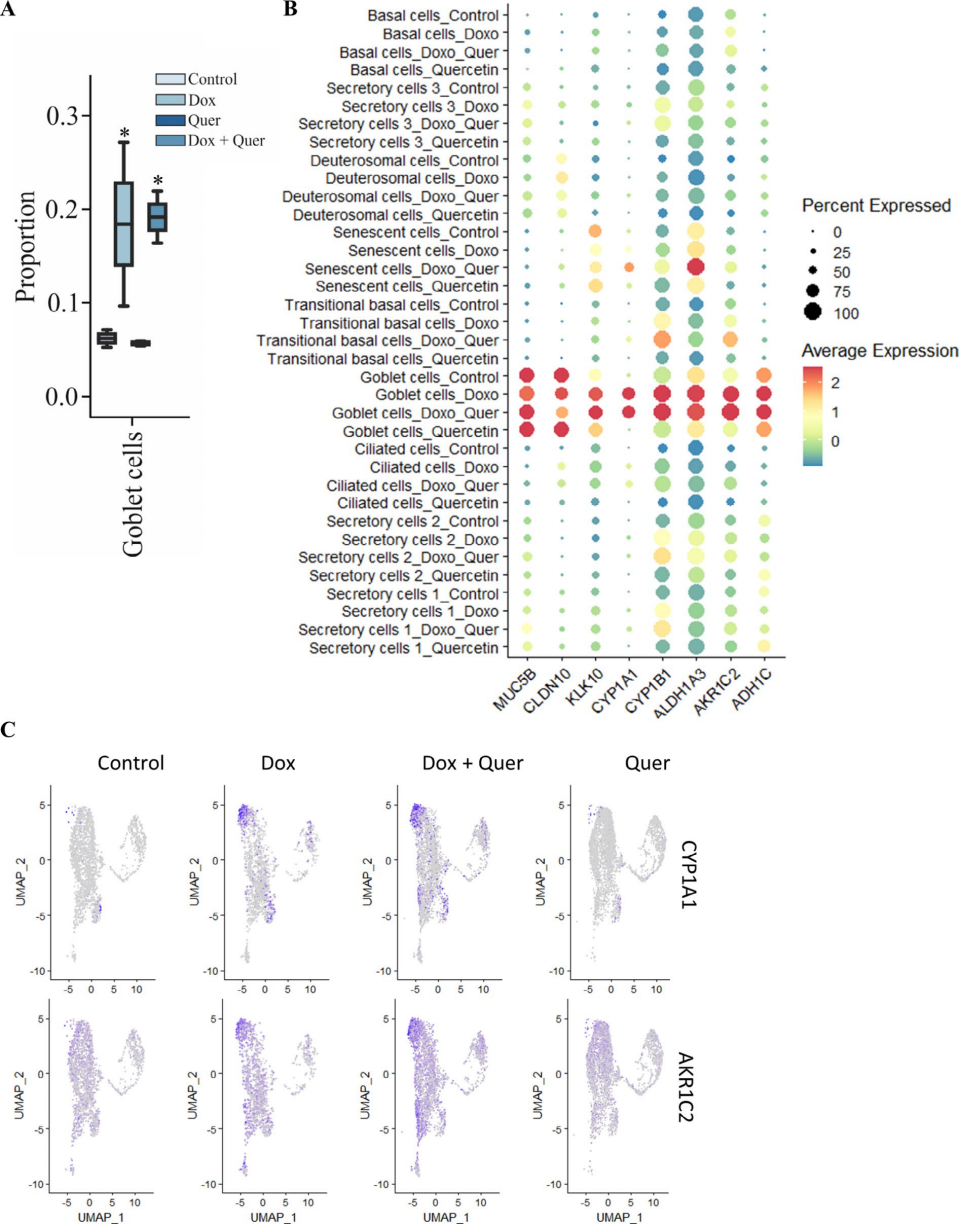
Doxorubicin induces the expression of detoxification factors in goblet cells. Organoids were pre-treated with Dox or control medium for 48 h. The organoids pre-incubated with control medium were treated with control medium or Quer, the organoids pre-incubated with Dox were treated with Dox or the combination of Quer and Dox for additional 48 h. (**A**) Proportion of the goblet cell cluster among all clusters. Data were compared by a Bayesian model. (**B**) Dot plot showing expression of goblet markers and factors associated with detoxification. (**C**) UMAP visualization of factors associated with detoxification

We calculated the proportion of different cell clusters for each donor (Fig. 5A). Treatment with Quer resulted in a significantly reduced proportion of senescent cells (Fig. 5B). Dox treatment also resulted in an increased proportion of goblet cells, which was not affected by the additional treatment with Quer (Fig. 6A). In addition, the expression of genes associated with detoxification (e.g. *CYP1A1*, *ALDH13A*) were strongly increased in the goblet cell cluster. Again, this was not affected by the Quer treatment (Fig. 6B and C).

### Senescence measurement in basal cells

**Fig. 7:**
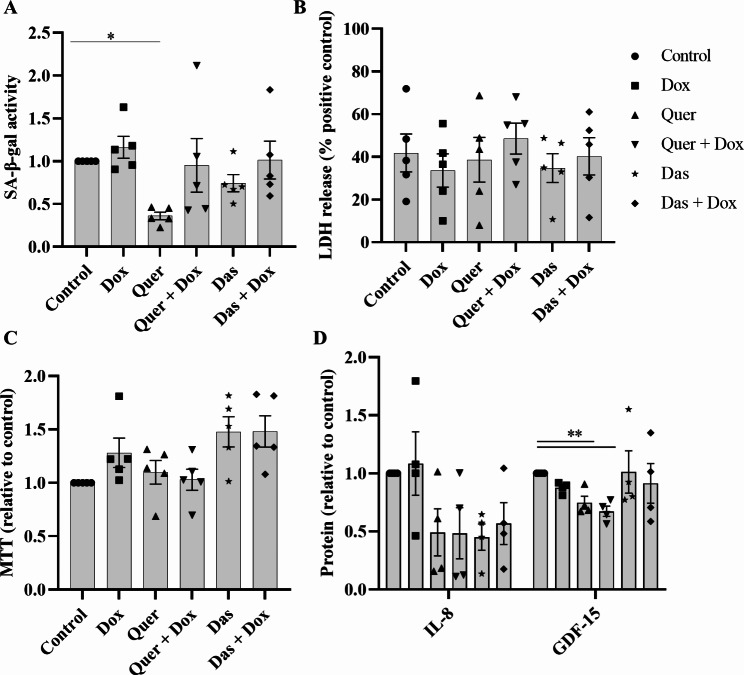
Senescence assay with non-differentiated basal cells. (**A**) SA-β-gal activity, (**B**) metabolic activity, (**C**) release of LDH, and (**D**) relative concentrations of GDF-15 and IL-8 in basal cell supernatants after treatment with Dox, Quer and Das and the combination of Dox and Quer or Das for 48 h. Each data point represents an independent experiment using cells from a separate donor. Data were compared by one-way ANOVA with Tukey’s multiple comparisons post-test and are shown as the mean ± SEM. *p < 0.05, **p < 0.01 and ***p < 0.001

We also tested whether Dox, Quer, or Das affect SA-β-gal activity in 2D-cultured basal cells. We treated basal cells cultured under conventional submerged conditions with Dox, Quer or Das as well as with the combination of Dox with Quer or Das for 48 h. Treatment with Dox did not significantly enhance SA-β-gal activity in basal cells, whereas Quer significantly decreased SA-β-gal activity, which was partially reversed by Dox (Fig. 7A). Das had no significant effect on SA-β-gal activity. The accumulation of LDH in supernatants did not significantly differ between the treatments (Fig. 7B). Treatment with the different compounds resulted in a slightly increased metabolic activity (Fig. 7C). Treatment with Quer, but not Das, resulted in significantly reduced release of GDF-15 (Fig. 7D).

## Discussion

**Fig. 8:**
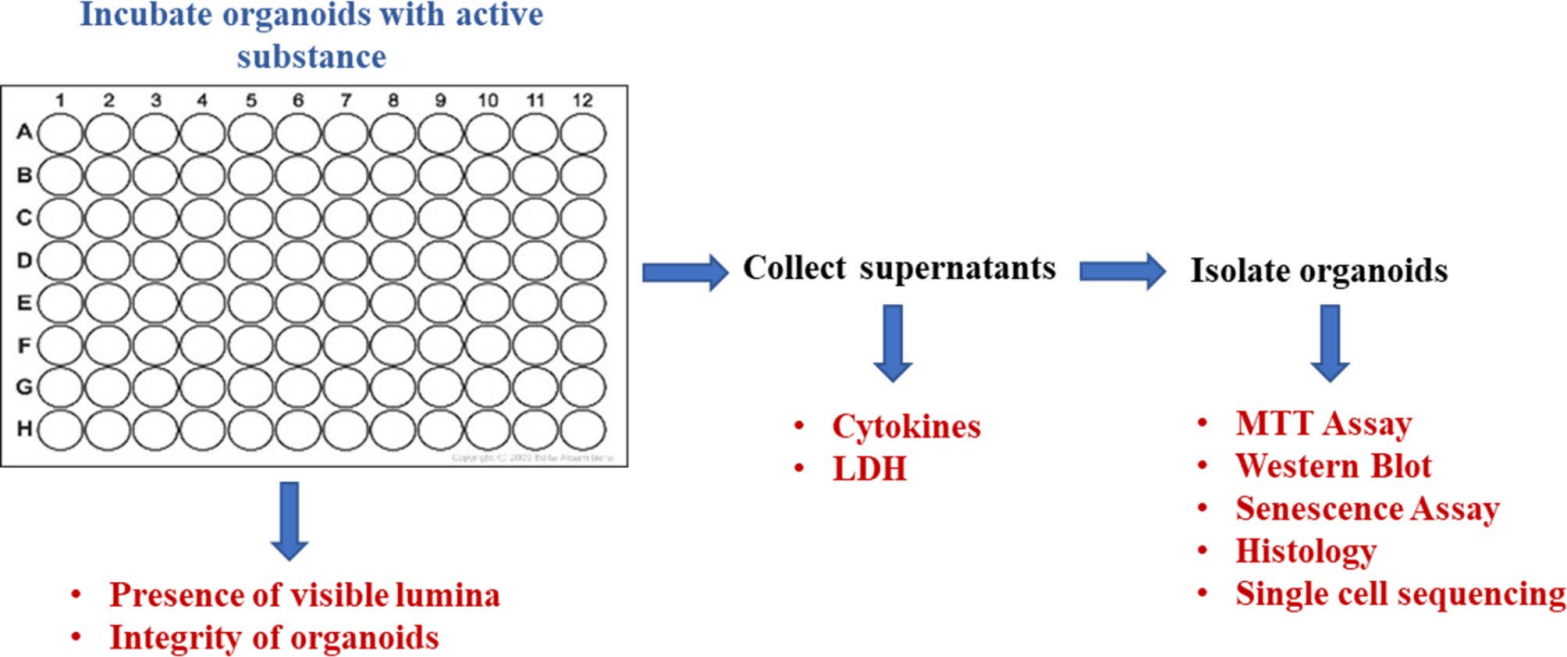
Scheme of the 3D lung organoid platform for pre-clinical testing of active substances. In the 96-well platform different endpoints can be measured in one assay run. Fluorescence measurements and microscopic images can be recorded directly in the plate. Various factors such as cytokines can be measured in the supernatants. Numerous methodologies such as biochemical assays and single cell sequencing can be carried out with isolated organoids

Here, we present a 3D lung organoid platform for the identification of active substances targeting epithelial senescence, toxicity, and inflammation under standardized conditions. In order to verify the platform, the extent to which the proposed senotherapeutic drugs like Quer and Das counteract Dox-induced senescence was tested in a series of independent experiments with organoids from different donors. To assess possible cytotoxic effects of the substances used, the integrity of the bronchospheres was documented and the metabolic activity as well as the release of LDH and cytokines were determined. The results showed that Quer and Das counteracted the senescence induced by Dox without cytotoxic effects. Analysis of supernatants also showed that Quer and Das selectively inhibit the release of epithelial mediators. Thus, the standardized testing of drugs in the 96-well platform is easy to handle and different endpoints can be measured in one assay run (Fig. 8).

In addition to the measurements that are easily carried out, organoids can be isolated for other endpoints, as demonstrated here for single cell sequencing and microscopic analysis. This is useful in identifying signaling pathways affected by selected tested drugs. The validation of the platform via single cell sequencing identified a cluster in which the cells expressed the senescence markers *CDKN1A1* (p21), *CDKN2A1* (p16), *GDF15* and *TIMP2* [29, 33]. Notably, this senescence cluster was the only one that was reduced by Quer treatment. This fits with the notion that Quer acts as a senolytic drug via selective removal of senescent cells [7]. However, it is also possible that Quer suppresses the induction of senescence or induces an alternative cell state which in this case would not be accompanied by elimination of senescent cells by apoptosis. Further studies are needed to elucidate the cellular mechanisms behind the effects of Quer.

In addition, our single cell data shows that Dox has a strong effect on goblet cells. Treatment with Dox resulted in increased expression of factors mediating detoxification and drug metabolism in goblet cells only (e.g. *CYP1A1, CYP1B1, ALDH1A3, AKR1C2, ADH1C*) [39, 40]. While Dox had no effect on the viability of the organoids, goblet cells were activated and their number increased proportionally. These data show very nicely that the different cell types of the epithelium react very differently to active substances. In our platform, active ingredients therefore show potentially acute adverse effects in specific cell types of a differentiated epithelium that cannot be shown in e.g. cell lines or classic 2D cultures with primary cells.

In a series of experiments, we further investigated the extent to which drugs can be tested for senescence using our assay with conventional 2D-cultured basal cells. The results obtained showed that undifferentiated basal cells provide insufficient information on how active substances act on a differentiated airway epithelium. No increased SA-β-gal activity could be determined after administration of Dox. However, Quer reduced the background senescence. This could be because senescent cells accumulate in conventional liquid culture [19] and are reduced by Quer as it has been shown for senescent human umbilical vein cells and bone marrow-derived murine mesenchymal stem cells before [28]. Furthermore, pure basal cells do not allow for a more in-depth analysis of the differential effects of novel substances on the different cell types of the airway epithelium, such as secretory and ciliated cells. Therefore, compared to 3D organoids, basal cells and cell lines (e.g. Calu-3, A549) are very limited for drug development, especially with regard to cellular signaling pathways and barrier formation.

Extensive in vivo studies with significant animal consumption are still performed in academic and industrial research, as well as in regulatory safety assessment and drug development [41]. In animal experiments investigating ​​the respiratory tract, mice are often exposed to noxae, and lung damage is induced by means of active substances or infection with pathogens. These models have the following serious disadvantages, among others: (i) significant animal consumption and animal suffering, (ii) differences in the physiology of the lungs between humans and rodents, and (iii) often insufficient transmissibility to humans due to differences in cellular processes between animals and humans [41, 42]. Moreover, according to the European animal protection law and additional regulations in many countries, animal experiments must be reduced to an essential and ethically justifiable level. It is therefore imperative to develop adequate models based on human cells in order to reduce the number of animals required in terms of the 3Rs (Replace, Reduce, Refine) and, in the best case, to replace them completely. Our platform meets these goals. It enables new active compounds and potential noxae to be tested not only with regard to senescence, but also inflammation and toxicity under standardized conditions.

In addition, we demonstrate that the system is also suitable for the analysis of cellular processes that are affected by identified active substances using modern methods such as single cell sequencing and common techniques such as fluorescence microscopy and histology. Such in-depth analyzes using human organoids are often more insightful than animal studies in terms of cellular mechanisms and the effect on humans, particularly when interspecies differences are considered [43]. In this way, the test system makes a significant contribution to an adequate selection of substances that are of interest for further preclinical evaluation and, if at all necessary, are further evaluated in animal experiments.

In summary, the 3D lung organoid platform allows for improved assessment and pre-selection of a wide range of compounds (e.g. natural products, pharmaceutically approved phytoextracts or antiphlogistics) in terms of senescence, inflammation and toxicity in preclinical studies. Through the use of differentiated human cells and modern methods for analyzing cellular signaling pathways, such platforms will increasingly replace animal experiments.

### Electronic supplementary material

Below is the link to the electronic supplementary material.

**Supplementary Material 1:** Supplemental information

## Data Availability

The datasets generated and analyzed during the current study are available from the corresponding author on reasonable request.

## Abbreviations

HBECs: Primary human epithelial cells
LDH: Lactate dehydrogenase
ELISA: Enzyme-linked immunosorbent assay
H&E: Hematoxylin-eosin
Dox: Doxorubicin
Quer: Quercetin
Das: Dasatinib
SA-β-gal: Senescence associated β-galactosidase
