# Supplementary material for: Biochemical and transcriptomic evaluation of a 3D lung organoid platform for pre-clinical testing of active substances targeting senescence
Source: Respir Res. 2024 Jan 3;25:3. doi: 10.1186/s12931-023-02636-7 (PMC10765931; doi:10.1186/s12931-023-02636-7)
Supplement: Supplementary file 1 — Supplementary Material 1: Supplemental information [file 12931_2023_2636_MOESM1_ESM.pdf]

Supplemental information
Methods

Western blot

For Western blot analysis, organoids were collected in RIPA Buffer (Cell Signaling Technology), supplemented with protease inhibitors (Complete Protease Inhibitor Cocktail Tablets, Roche Diagnostics, Switzerland). The amount of total protein was determined with Pierce BCA-Protein Assay Kit (Thermo Fisher, USA). Samples were denaturated and separated on a 12% SDS polyacrylamide gel. Proteins were transferred to a nitrocellulose membrane, and membranes were probed against p21 (p21 (1/1000 diluted, Waf1/Cip1). The blots were stripped and reprobed for ß-actin (1/1000 diluted, Nr. 4967, Cell Signaling Technology). Signals were detected by enhanced chemiluminescence (BioRad, Dreieich, Germany) using appropriate peroxidase-conjugated secondary antibodies (Agilent, DAKO, Santa Clara, CA, USA).

Histology/microscopy

Organoids from each group were pooled and embedded in paraffin as described before [1]. Sections of 2 µm thickness were stained with hematoxylin-eosin (H&E). Deparaffinized paraffin sections were treated with BSA (1%) and Tween-20 in PBS (0.1%) and incubated overnight at 4 °C with the primary antibodies for p21 (1:100, Cell signaling (2947S)) and TIMP2 (1:400, Invitrogen (MA1-774)) in PBS containing BSA (1%). Cells were incubated for 30 min with secondary antibodies (goat anti-rabbit FITC, Sigma; goat anti-mouse, Cyanine 5, Thermo-Fisher Scientific). ImageJ software (National Institutes of Health) was used to analyze and merge the images.

Senescence Assay (Flow Cytometry)

Organoids from two donors were treated with Dox or control media and dissociated as described in the single cell section. Activity of ß-galactosidase was measured using the Senescence Assay Kit (Abcam, UK) according to the manufacturer's protocol.

Supplementary figures

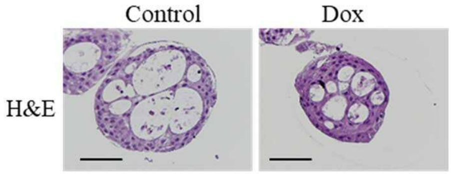

S1: Representative histology of embedded organoids (H&E staining, scale bar = 50 µm).

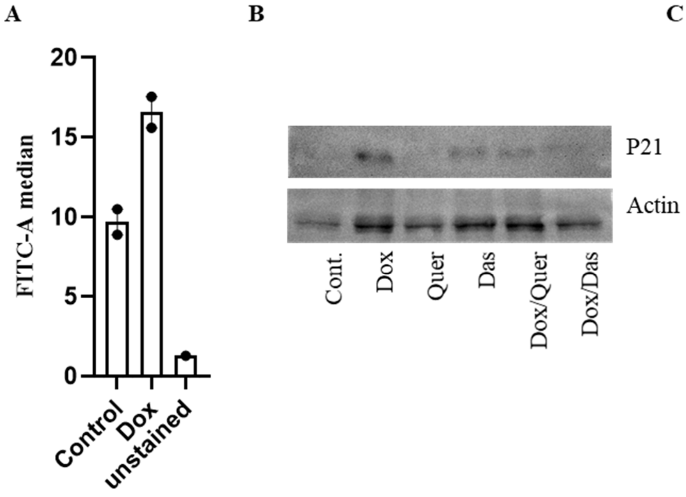

S2: (A) Organoids from two donors were treated with Dox for 48 hours and SA-β-gal activity was measured by flow cytometry. (B/C) Western blot analysis for p21. Organoids were treated with Dox and the combination of Dox with Quer or Das for 48 hours. (B) Cell lysates were gel separated and immunoblotted. (C) Semi-quantitative densitometry normalized to actin.

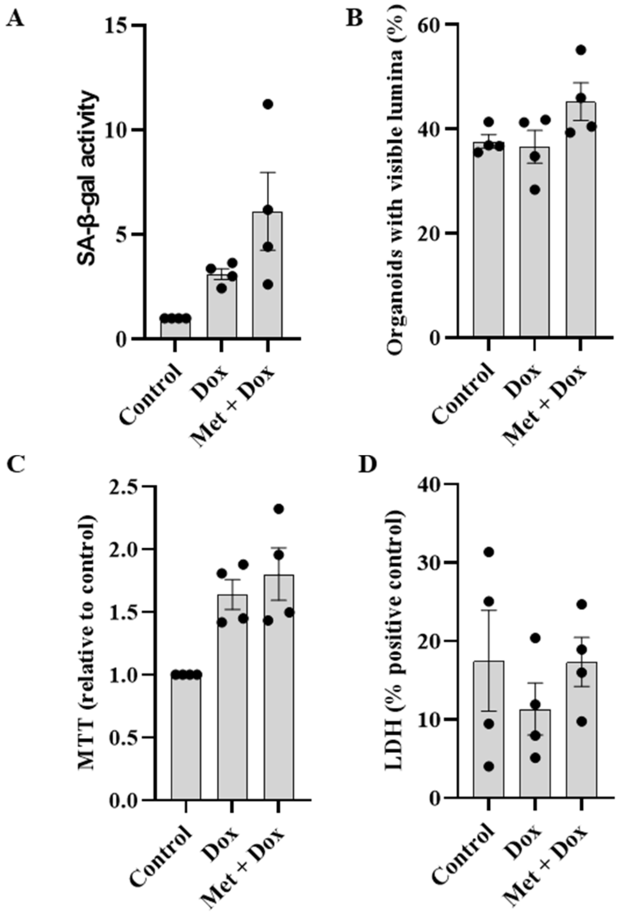

S3: Metformin does not inhibit senescence in organoids. (A) SA-β-gal activity, (B) percentage of organoids with visible lumen, (C) metabolic activity, and (D) release of LDH after 48 hours treatment of organoids with Dox and the combination of Dox with Met. Each data point represents an independent experiment. Data are shown as the mean ± SEM.

A

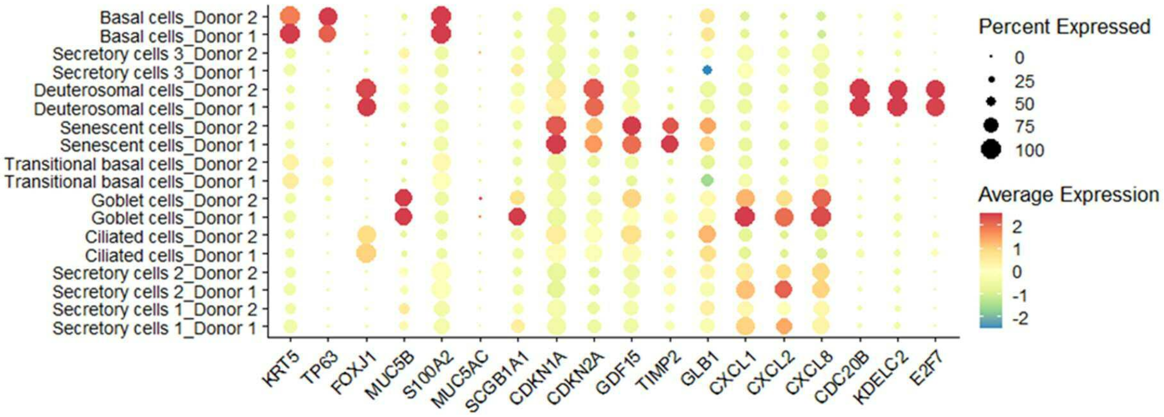

Basal cell marker

B

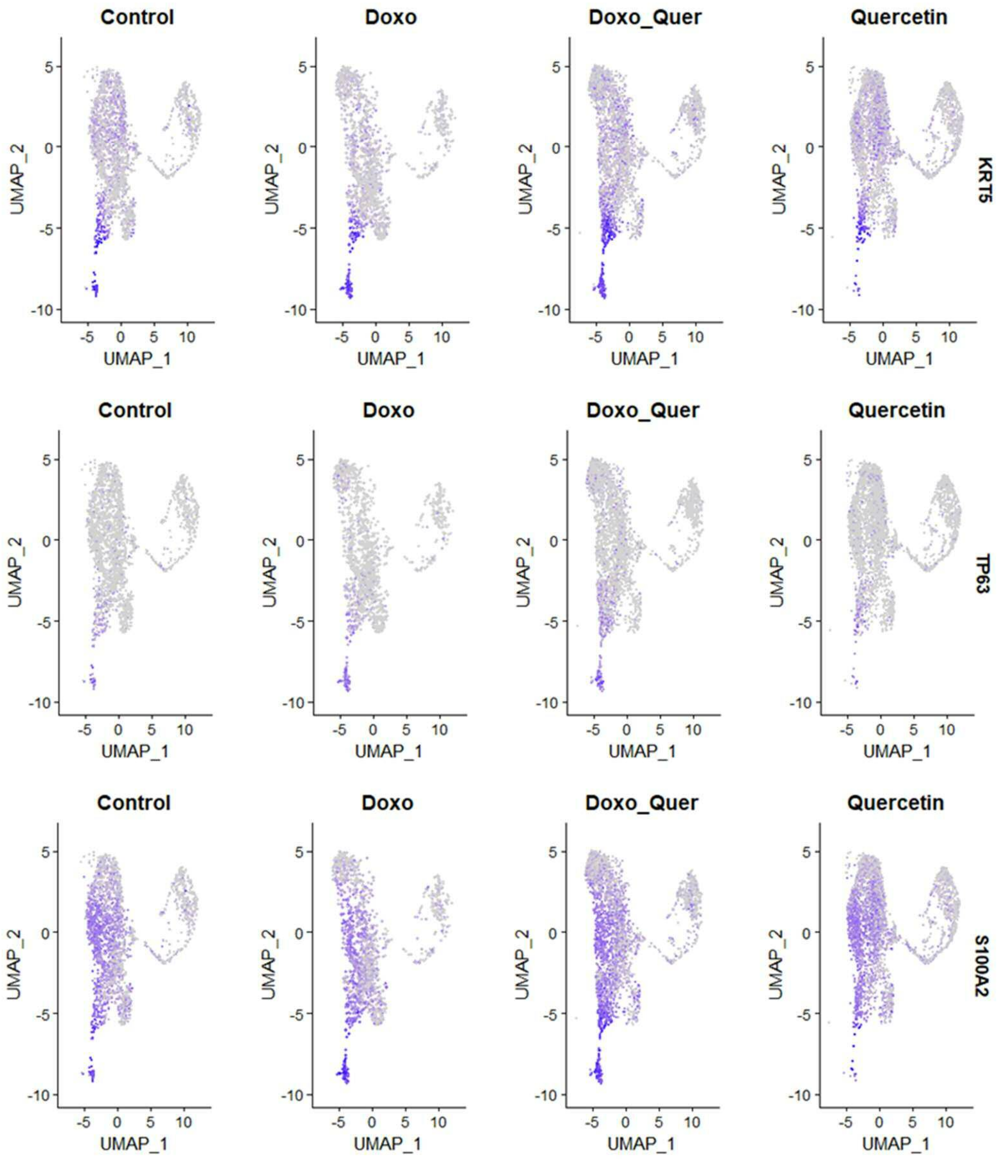

Secretory marker

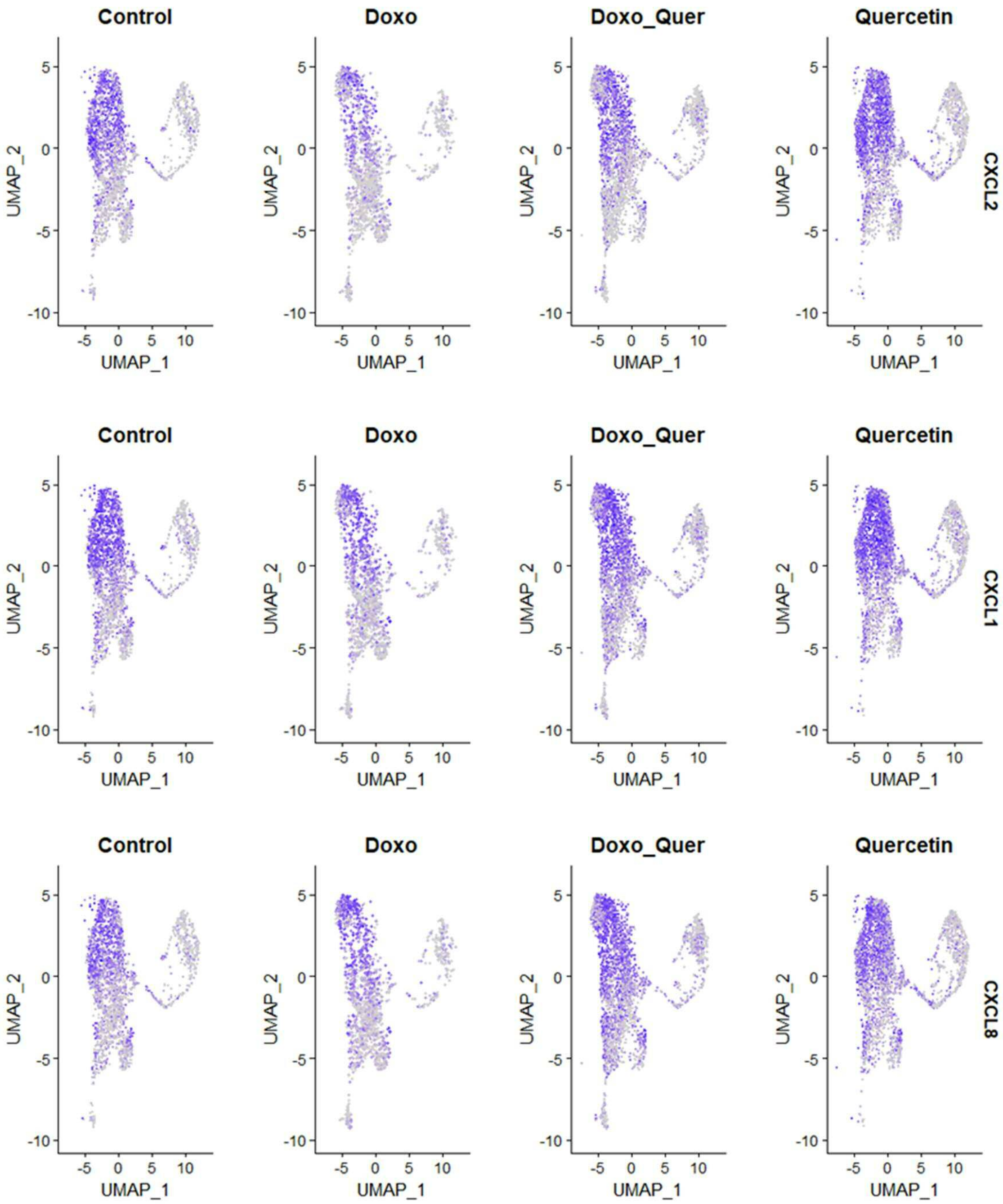

Goblet cell marker

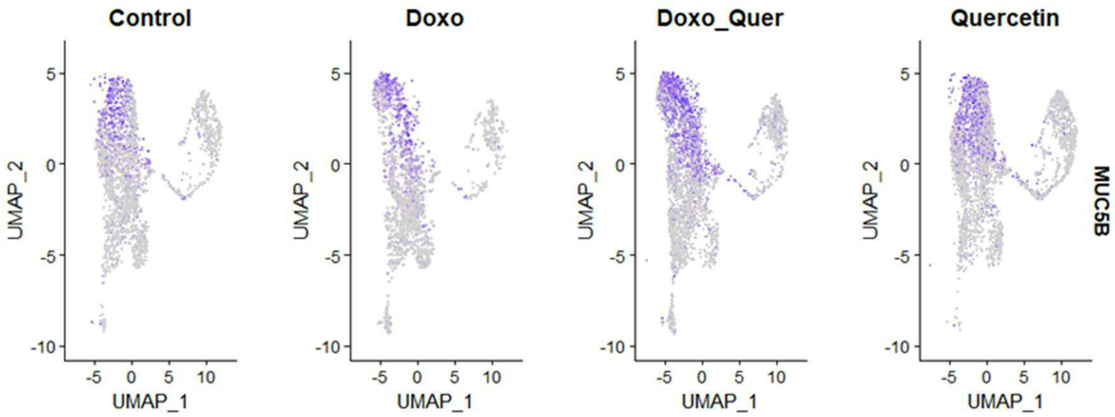

Ciliated cell marker

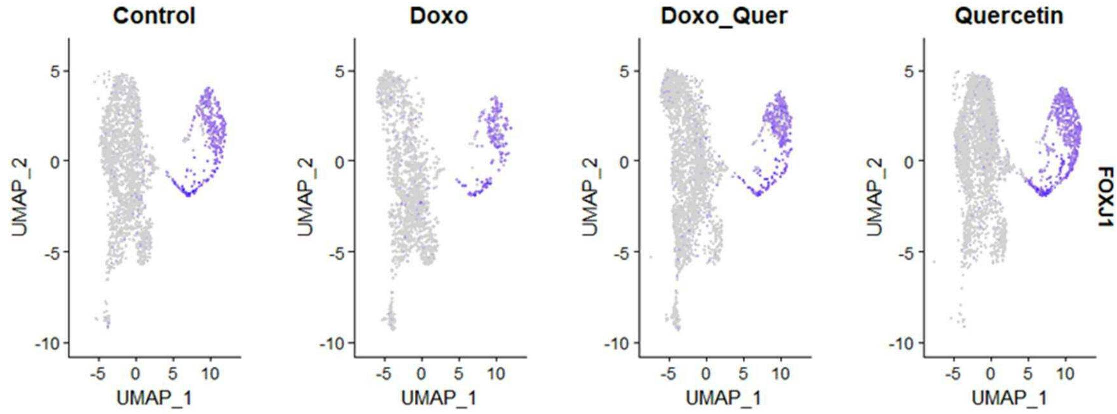

Senescence marker

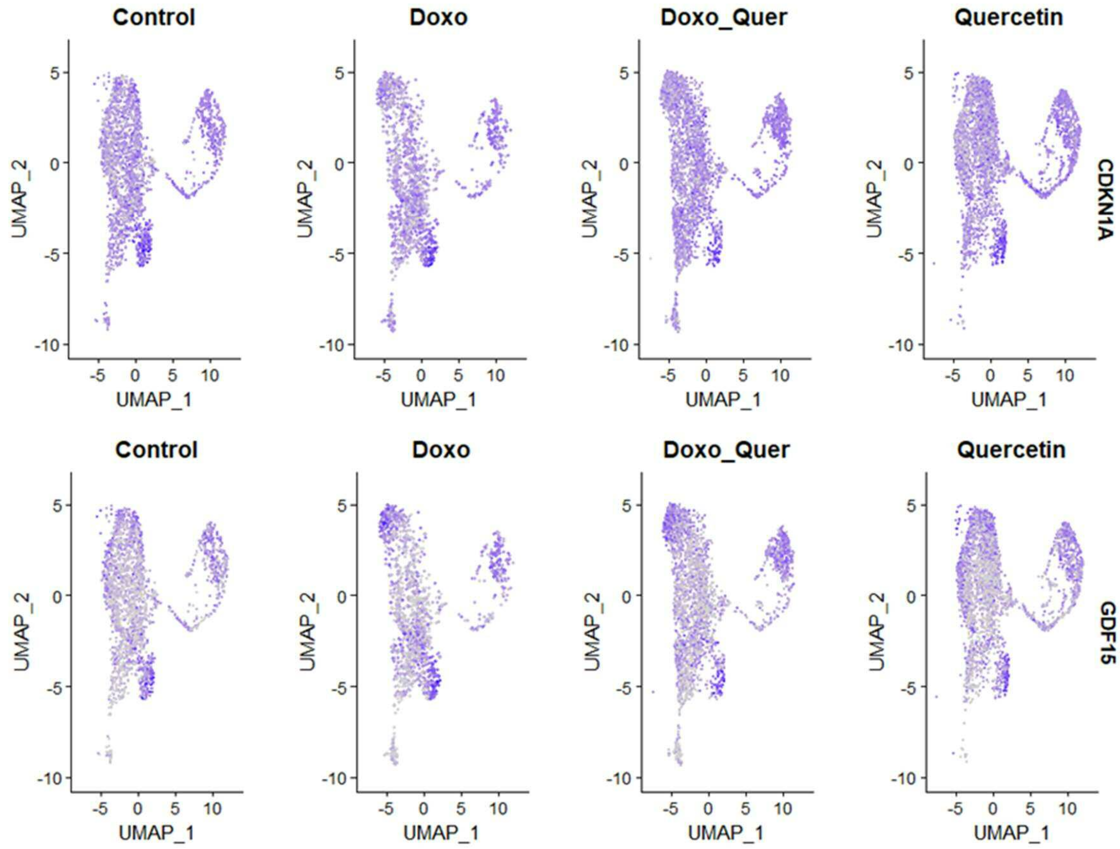

S4: Organoids were pre-treated with Dox or control medium for 48 hours. The organoids pre-incubated with control medium were treated with control medium or Quer, the organoids pre-incubated with Dox were treated with Dox or the combination of Quer and Dox for additional 48 hours. (A) Dot plot showing expression of epithelial cell type markers. (B) UMAP visualization of cell markers.

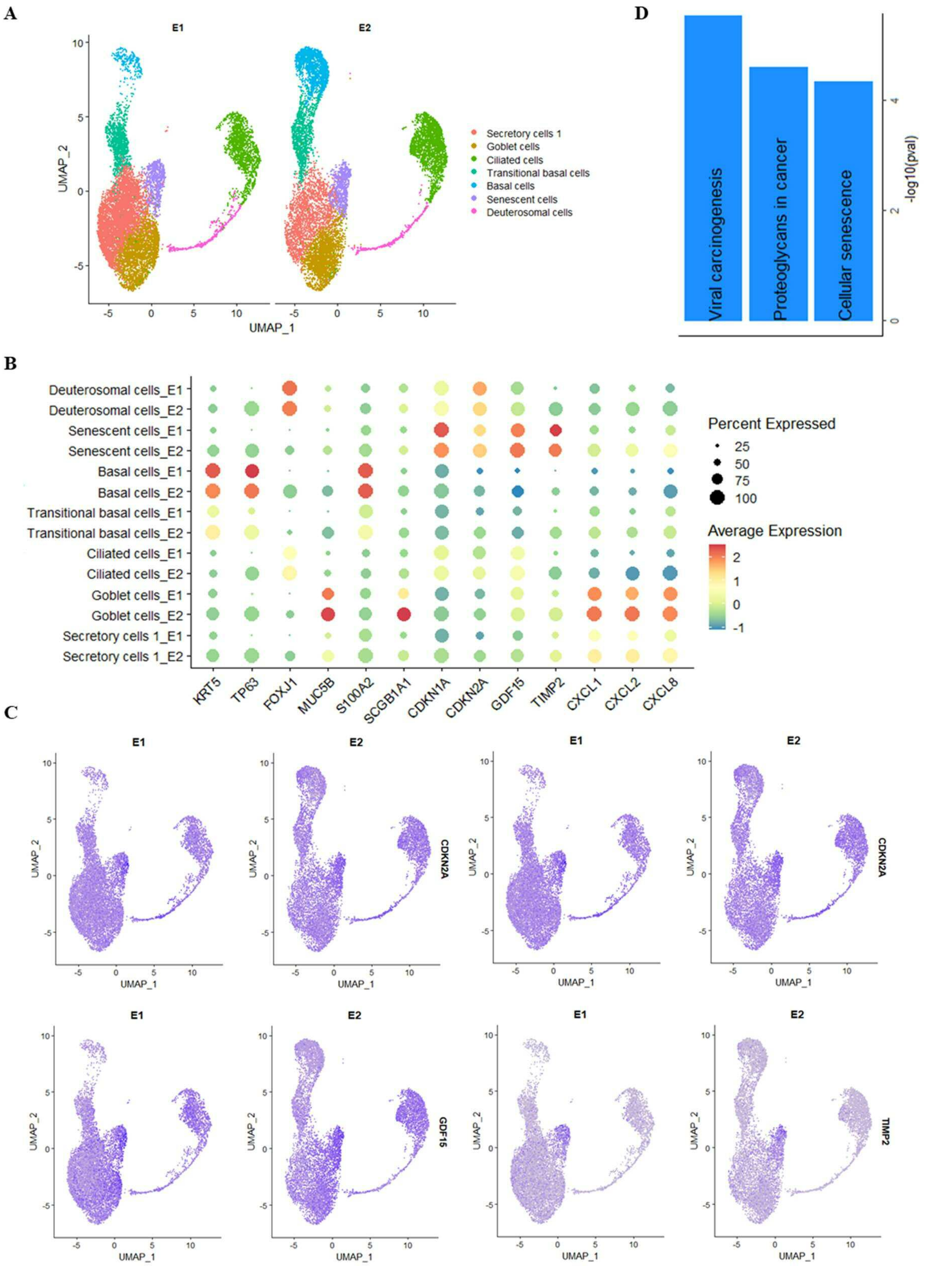

S5: The single cell data (E1) were combined with an independent data set containing untreated organoids from 5 donors (E2). (A) UMAP visualization of major epithelial cell types of combined data sets. (B) Dot plot showing expression of epithelial cell type markers. (C) UMAP visualization of cell markers.

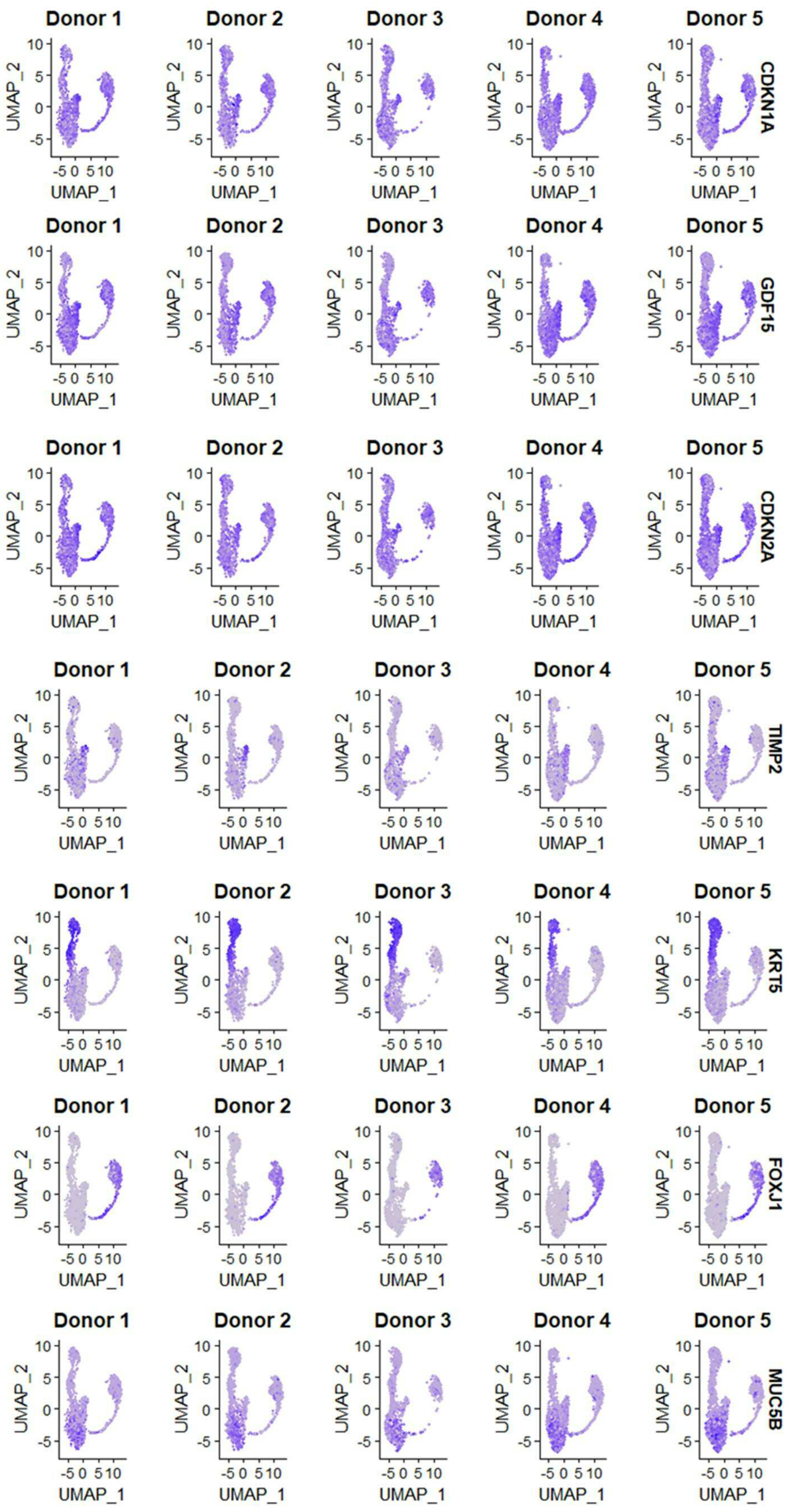

S6: The single cell data were combined with an independent data set containing untreated organoids from 5 donors. UMAP visualization of cell markers for the 5 donors

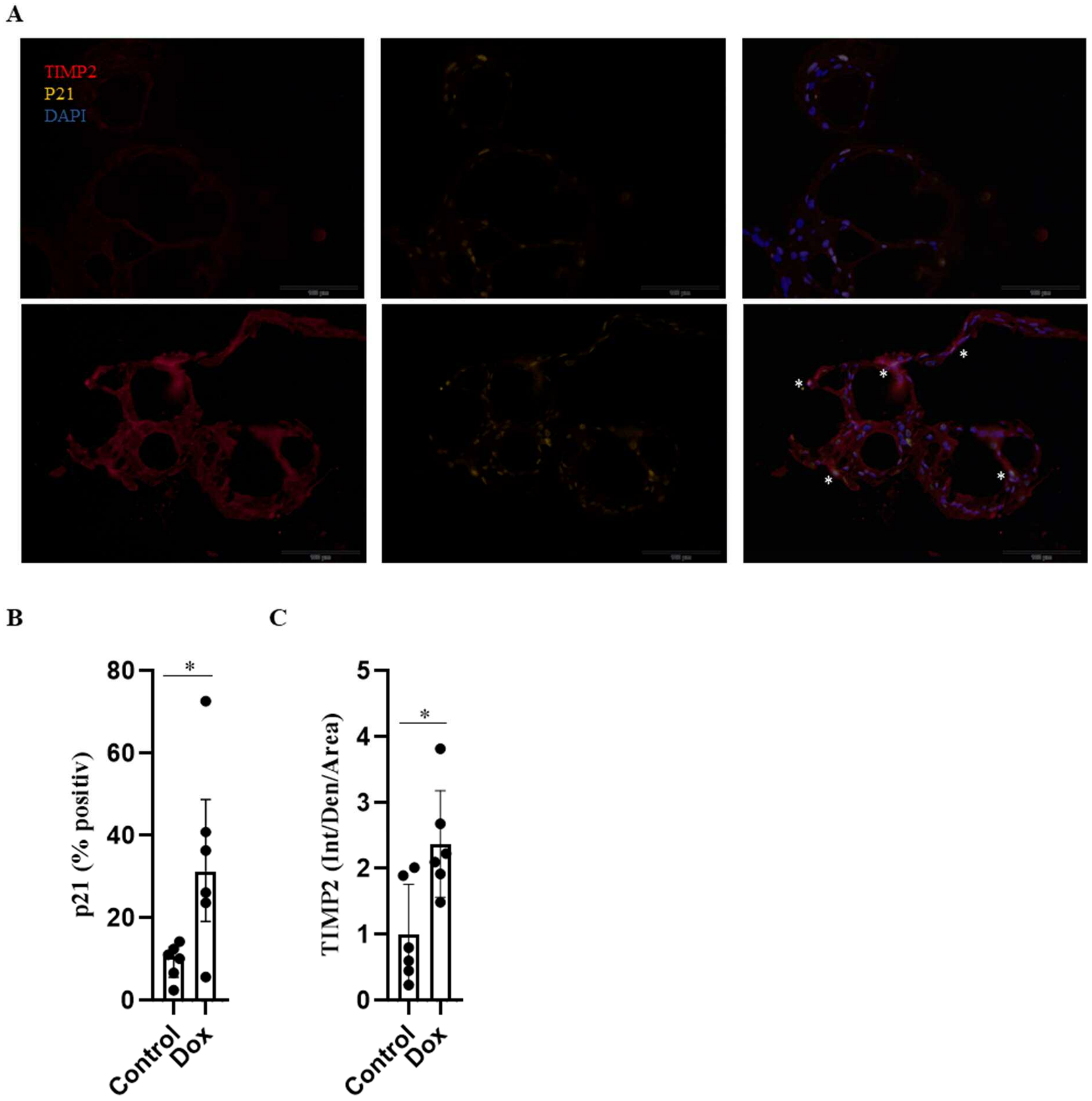

S7: Organoids were treated with Dox or control media for 48 hours. (A) Immunofluorescence staining was performed for TIMP2 (red) and p21 (green yellow). Nuclei were stained with DAPI (blue). Scale bar: 100 µm. * indicate increased staining for TIMP2 associated with p21 positive nuclei. Quantification of (B) p21-positive nuclei and (C) TIMP2. Pooled results from two donors. Data were compared by Mann Whitney test and are shown as the median with interquartile range. $p < 0.05$.

A

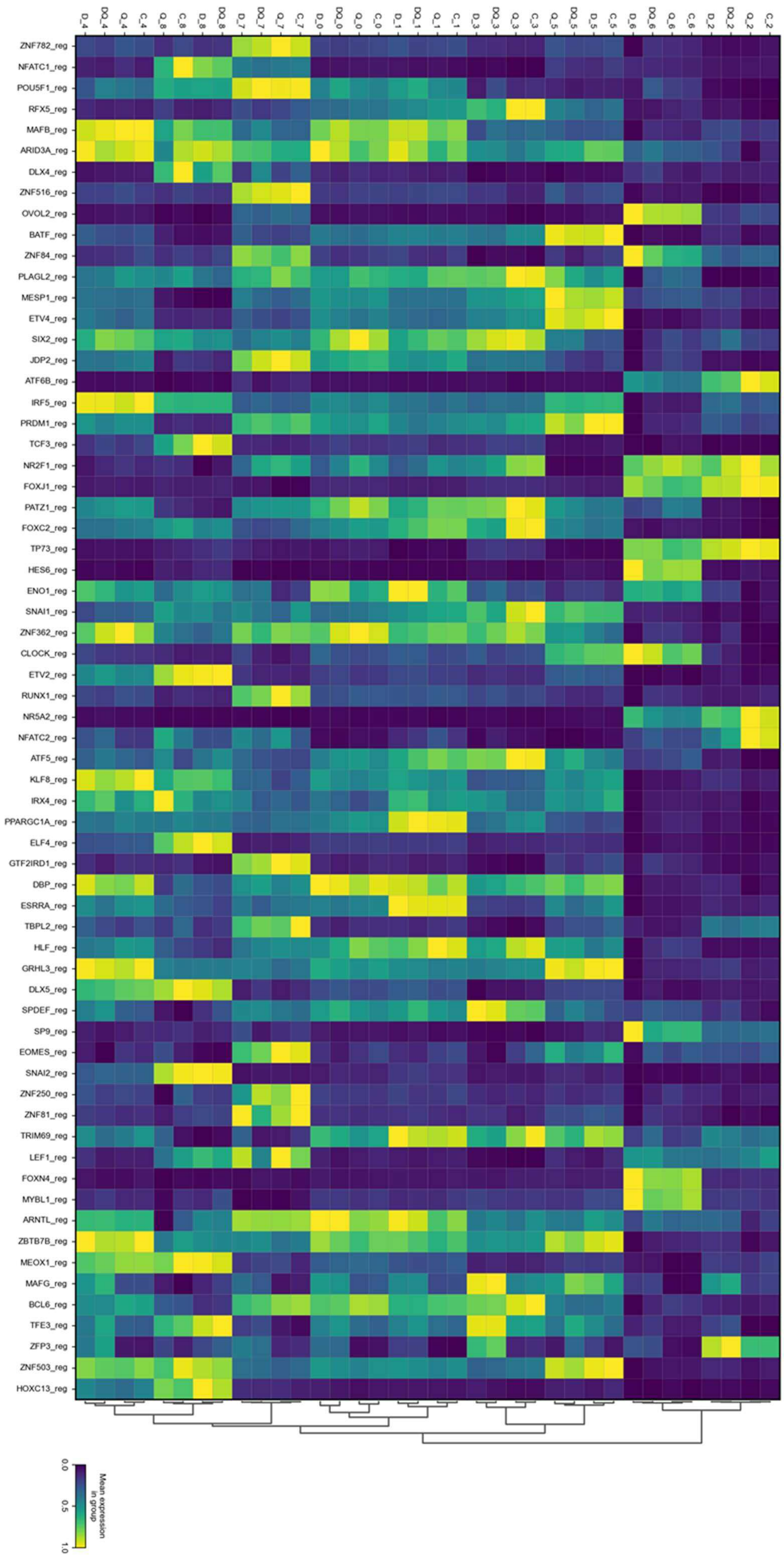
D_4
DQ_4
Q_4
C_4
Q_8
C_8
D_8
DQ_8
D_7
DQ_7
Q_7
C_7
D_0
DQ_0
Q_0
C_0
D_1
DQ_1
Q_1
C_1
D_3
DQ_3
Q_3
C_3
Q_5
DQ_5
D_5
C_5
D_6
DQ_6
Q_6
C_6
D_2
DQ_2
Q_2
C_2
ZNF782_reg
NFATC1_reg
POU5F1_reg
RFX5_reg
MAFB_reg
ARID3A_reg
DLX4_reg
ZNF516_reg
OVOL2_reg
BATF_reg
ZNF84_reg
PLAGL2_reg
MESP1_reg
ETV4_reg
SIX2_reg
JDP2_reg
ATF6B_reg
IRF5_reg
PRDM1_reg
TCF3_reg
NR2F1_reg
FOXJ1_reg
PATZ1_reg
FOXC2_reg
TP73_reg
HES6_reg
ENO1_reg
SNAI1_reg
ZNF362_reg
CLOCK_reg
ETV2_reg
RUNX1_reg
NR5A2_reg
NFATC2_reg
ATF5_reg
KLF8_reg
IRX4_reg
PPARGC1A_reg
ELF4_reg
GTF2IRD1_reg
DBP_reg
ESRRA_reg
TBPL2_reg
HLF_reg
GRHL3_reg
DLX5_reg
SPDEF_reg
SP9_reg
EOMES_reg
SNAI2_reg
ZNF250_reg
ZNF81_reg
TRIM69_reg
LEF1_reg
FOXN4_reg
MYBL1_reg
ARNTL_reg
ZBTB7B_reg
MEOX1_reg
MAFG_reg
BCL6_reg
TFE3_reg
ZFP3_reg
ZNF503_reg
HOXC13_reg
Mean expression in group
0.0
0.5
1.0

B

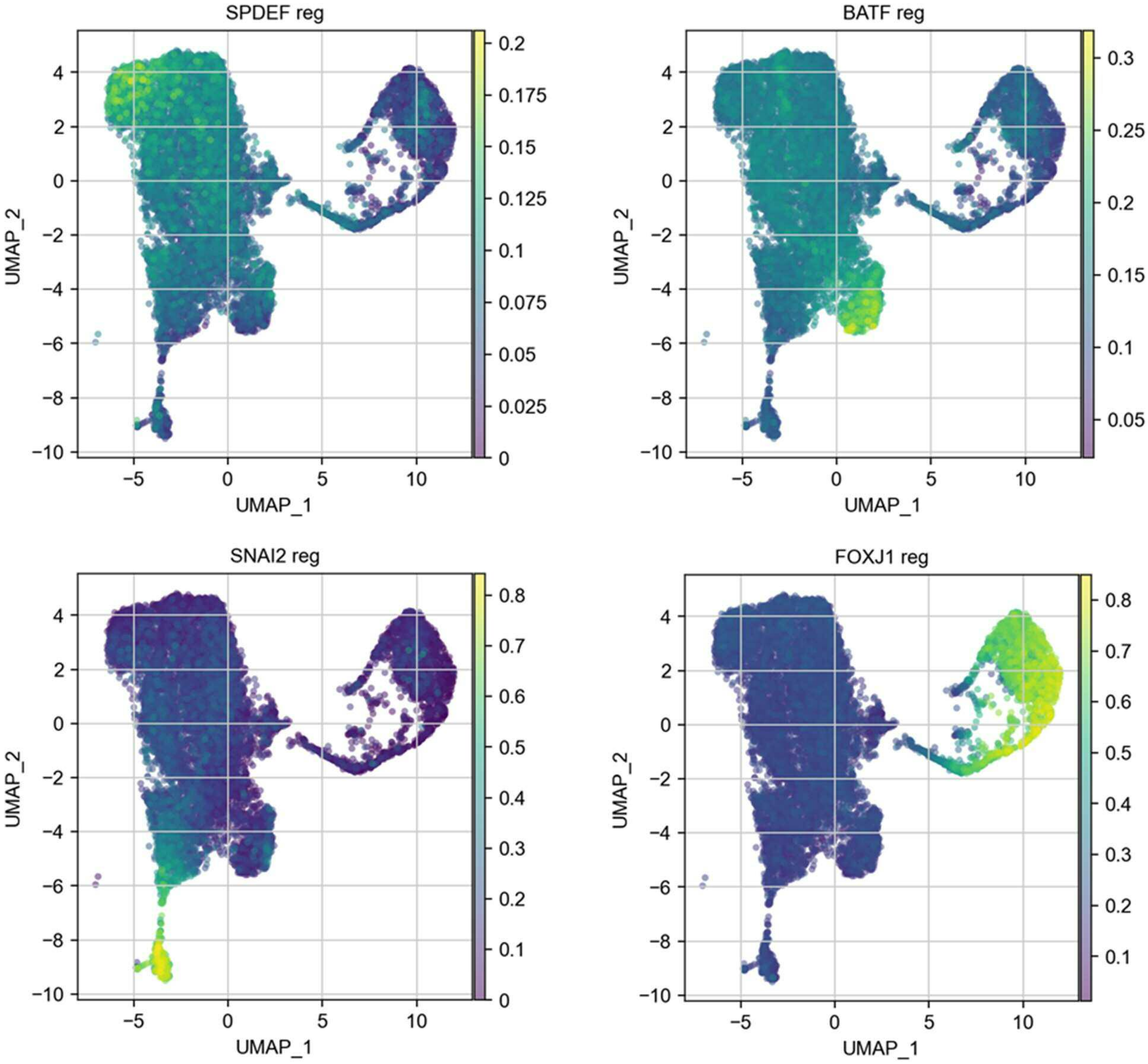

S8: Organoids were pre-treated with Dox or control medium for 48 hours. The organoids pre-incubated with control medium were treated with control medium or Quer, the organoids pre-incubated with Dox were treated with Dox or the combination of Quer and Dox for additional 48 hours. (A) Heatmap of regulon activity of the top regulons discriminating the clusters. C, control media; D, Dox media; CQ, control media with Quer; DQ, Dox/Quer media. (C) UMAP representations.

1. Sprott RF, Ritzmann F, Langer F, Yao Y, Herr C, Kohl Y, Tschernig T, Bals R, Beisswenger C: **Flagellin shifts 3D bronchospheres towards mucus hyperproduction.** *Respir Res* 2020, **21:**222.
